# High-Dose Amoxicillin with Clavulanate for the Treatment of Acute Otitis Media in Children

**DOI:** 10.1155/2014/965096

**Published:** 2014-01-06

**Authors:** Chia-Huei Chu, Mao-Che Wang, Liang-Yu Lin, Tzong-Yang Tu, Chii-Yuan Huang, Wen-Huei Liao, Ching-Yin Ho, An-Suey Shiao

**Affiliations:** ^1^Department of Otorhinolaryngology-Head and Neck Surgery, Taipei Veterans General Hospital, No. 201, Section 2, Shih-Pai Road, Taipei 11217, Taiwan; ^2^Department of Otorhinolaryngology, National Yang-Ming University School of Medicine, No. 155, Section 2, Li-Nong Street, Taipei 11217, Taiwan; ^3^Division of Endocrinology and Metabolism, Department of Medicine, Taipei Veterans General Hospital, No. 201, Section 2, Shih-Pai Road, Taipei 11217, Taiwan; ^4^Department of Medicine, National Yang-Ming University School of Medicine, No. 155, Section 2, Li-Nong Street, Taipei 11172, Taiwan; ^5^Institute of Pharmacology, National Yang-Ming University School of Medicine, No. 155, Section 2, Li-Nong Street, Taipei 11217, Taiwan

## Abstract

*Objective*. This study uses the acute otitis media clinical practice guideline proposed in 2004 as a reference to evaluate whether antibiotics doses that are in line with the recommendations lead to better prognosis. The study also attempts to clarify possible factors that influence the outcome. *Study Design*. Retrospective cohort study. *Subjects and Methods*. A total of 400 children with acute otitis media were enrolled. The dosage of amoxicillin was considered to be appropriate when in accord with clinical practice guidelines, that is, 80–90 mg/kg/day. The outcome was defined according to the description of tympanic membrane on medical records. Multivariate logistic regression was used to analyze the relationship between antibiotic dosage and prognosis after adjusting for baseline factors. *Results*. The majority of prescriptions were under dosage (89.1%) but it was not noticeably associated with outcome (*P* = 0.41). The correlation between under dosage and poor prognosis was significant in children below 20 kg with bilateral acute otitis media (odds ratio 1.63; 95% CI 1.02–2.59, *P* = 0.04). *Conclusion*. Treating acute otitis media in children, high-dose amoxicillin with clavulanate as recommended in the clinical practice guideline was superior to conventional doses only in children under 20 kg with bilateral diseases.

## 1. Introduction

Acute otitis media (AOM) is one of the common childhood infections, occurring often after acute upper respiratory tract infections. It is also the leading cause of clinic visits by children and the most frequent reason that doctors prescribe antibiotics [1–3]. According to insurance statistics, AOM-related outpatient services in the US were as high as 16 million in 2000, and 80% of the cases were prescribed with antibiotics. It had been reported that the estimated annual medical expenditure related to AOM is about $3.8 billion to $5.3 billion in the US [4, 5]. The total cost per AOM episode ranged from €332.00 to €752.49 in several European countries [6]. In addition to medical expense from physicians' consultation and medications, AOM indirectly causes the loss of children's school time and caregivers' work time and income [7].

To reduce medical costs while maintaining high standards of medical quality, the American Academy of Pediatrics, the American Academy of Otolaryngology-Head and Neck Surgery, and the American Academy of Family Physicians endorsed an evidence-based comprehensive review, the AOM Clinical Practice Guidelines: Diagnosis and Management of AOM, which was published in May 2004 [8]. The guidelines emphasize the importance of correct diagnosis and provide suggestions for initial treatment of children between the age of 2 months and 12 years with uncomplicated AOM.

Amoxicillin with or without *β*-lactamase inhibitor is the mainstream choice in the treatment of AOM, but information on dosage comparisons of antibiotics and research on true clinical outcome is limited [9–11]. In the past, most studies on AOM focused on epidemiological surveys, laboratory bacteriological reports, and the therapeutic effects of different antibiotics [12, 13]. Experts have published different viewpoints on the suggestions in the clinical practice guidelines, such as when to provide antibiotic treatment, how to give an adequate dose, and the proper duration of treatment. As most of the evidence comes from bacteriological research in laboratories and a small number of samples in clinical studies, higher level evidence is lacking [14–17]. Therefore, doctors still do not have a consensus on these issues [18].

This study aimed to evaluate whether physicians' prescriptions are in accordance with the dosage guidelines and whether high-dose amoxicillin with clavulanate is associated with better outcomes in pediatric AOM.

## 2. Subjects and Methods

### 2.1. Subjects

We conducted a retrospective cohort study from January 1, 2005, to December 31, 2008. Children aged 2 months to 12 years who were diagnosed with acute otitis media according to the diagnosis code (382.00) in the International Classification of Diseases, Ninth Revision, Clinical Modification (ICD-9-CM), were enrolled. Those who had been coded with any anatomic or genetic abnormalities, such as craniofacial anomalies or Down syndrome, or immune deficiencies were excluded. Children with histories of recurrent AOM (three or more previous episodes of AOM within 12 months), those who had undergone any middle ear or inner ear procedure, those who had only one visit, or those missing records were excluded. Their age, gender, weight, diagnosis date, unilateral or bilateral disease, and details in antibiotic prescriptions were reviewed.

### 2.2. Methods

Antibiotics prescriptions that contained amoxicillin with clavulanate (amoxicillin 400 mg with clavulanate 57 mg/5 mL) were reviewed in detail. Dosage was considered to be correct, that is, high dose, if the amoxicillin component was within ±10% of the guideline suggestions. If the dosage was outside the ±10% range, it was considered either over- or under dose. The recommended dose of the amoxicillin component is 80–90 mg/kg/day. However, for children over 20 kg, this might correspond to a dosage that surpasses the common clinical dosage for adults of 1500 mg/day. Therefore, a daily dosage of 1500 mg amoxicillin for children over 20 kg was considered to be acceptable.

To evaluate the relationship between amoxicillin dose and AOM prognosis, study analysis focused on the comparison of two groups: those who were prescribed with an amoxicillin dose that was in agreement with the guideline recommendations (80–90 mg/kg/day) those who were not.

The assessment of treatment outcome was based on medical records within 14 days after the antibiotic prescription expiry date. In our institute, the middle ear status in children was mainly evaluated by telescope examination by a licensed otology specialist which had been demonstrated to have the highest sensitivity (97.8%) and specificity (100.0%) [19]. Successful control was defined as a medical record of an eardrum that either was normal or showed otitis media with effusion (OME). Failed control was defined as improvement in only one of two affected ears or a change in antibiotics before the end of the treatment period (with the reason for changing antibiotics being failure to control illness rather than side effects).

## 3. Statistical Analysis

Continuous variables such as age and body weight are presented as mean and standard deviation. Gender, single-ear or bilateral disease, and illness season were categorical variables and represented as numbers and percentages. To analyze the possible prognostic factors, we used a *t*-test and chi-square test for univariate analysis. All covariates in univariate analyses along with the two basic demographic variables (gender and weight) were then included in a binary logistic regression model. All statistical analyses were carried out with IBM SPSS statistical software version 14.0 for Windows (IBM Corp., New York, USA). Statistical significance was defined as a *P* value of less than 0.05.

## 4. Results

### 4.1. Clinical Characteristics of the Study Population

A total of 400 medical records with diagnosis code 382.00 were reviewed. There were 94 children with complicated AOM (i.e., clinical recurrence of AOM within 30 days, histories of recurrent AOM, AOM with underlying chronic otitis media with effusion, any underling anomalies that may alter the course of AOM such as cleft palate, genetic problems such as Down syndrome, immunodeficiencies, and status after any middle or inner ear surgery); 59 had only one visit, 40 had incomplete record, 15 were prescribed with cephalosporin or sulfa drugs, and 27 were prescribed with amoxicillin. One hundred and sixty five children treated with amoxicillin with clavulanate were included into analysis. The average age of the 165 enrolled patients was 4.91 years old (0.28–11.72 years old), the average weight was 19.36 kg (7.50–48.0 kg), and 94 were boys (57%). Most illnesses occurred in spring (31.5%) and autumn (28.5%). Single-ear AOM occurred in 81 participants (49.1%) (Table 1).

### 4.2. Antibiotic Dosage

Eighteen in 165 (10.9%) prescriptions were in accord with the recommendations of the clinical practice guidelines. Underdosage was extremely common (89.1%). None of the prescriptions exceeded the upper limit of 90 mg/kg/day. Overall, the average dose of the amoxicillin component was 45.5 mg/kg/day, which was far lower than the guideline suggestion. However, 86 (52.1%) prescriptions were within the range of 40–50 mg/kg/day, the traditional usage for amoxicillin. In other words, half of the physicians indeed followed the conventional dosage.

### 4.3. Antibiotic Dosage and Treatment Outcome

Successful control was achieved in 121 patients (73.3%) while the remaining 26.7% had failed control. Fewer patients who were given the correct dose (i.e., high-dose amoxicillin) had poor AOM prognosis (16.7% versus 27.9%), but this result was not significant (Fisher exact test, *P* = 0.41) (Table 2).

### 4.4. Factors Affecting Treatment Outcome

In univariate analysis, only disease in autumn/winter was associated with a poor prognosis (*P* = 0.03). Age (*P* = 0.22), gender (*P* = 0.07), and body weight (*P* = 0.25) were not associated with treatment outcome. Bilateral AOM was borderline significantly correlated with poor disease control (*P* = 0.05).

Since antibiotics prescribed in children are based on body weight, the age and weight are highly correlated (Spearman's rho = 0.88, *P* < 0.001); thus body weight was chosen instead of age in the following analyses. Using multivariate analysis, we analyzed relevant factors that influenced AOM prognosis (Table 3); it is found that, compared to patients with AOM in the spring/summer, the odds ratio (OR) of autumn/winter patients who failed control was 2.47 (95% confidence interval (CI): 1.17–5.23, *P* = 0.02).

### 4.5. Subgroup Analysis

According to the newest international growth standards for infants and children from the World Health Organization (2007) [20], with 50% percentile estimation, children weigh about 20 kg at 6 years of age. Since the daily dosage for children over 20 kg might surpass the equivalent common clinical dosage for adults of 1500 mg/day, we performed subgroup analysis for 110 children who weighed less than 20 kg (details not shown). Among this subgroup, illness in autumn/winter was consistently a strong factor for poor prognosis (OR 4.80; 95% CI 1.82–12.67, *P* = 0.002). In addition, the risk of failed control in bilateral AOM patients was 2.43 times more than single-sided AOM children although it was not statistically significant (95% CI 0.90–6.53, *P* = 0.08). No obvious association was observed between under dose and AOM prognosis (OR 2.18; 95% CI 0.38–12.48, *P* = 0.38).

When “bilateral AOM” and “under dose” were taken together, their correlation with treatment failure was evident (OR 1.63; 95% CI 1.02–2.59, *P* = 0.04). Again, AOM in autumn/winter was consistently associated with a poor prognosis (OR 4.90; 95% CI 1.87–12.89, *P* = 0.001). The results implied that the benefit of high-dose amoxicillin with clavulanate as recommended in the guidelines was more noticeable in children under 20 kg with bilateral AOM (Table 4).

## 5. Discussion

Our study found no significant correlation between high-dose amoxicillin and better disease control when treating AOM with amoxicillin-clavulanate combination. Although fewer patients who were given the high dosage failed to control AOM (16.7% for high dose versus 27.9% for under dose), the correlation was not evident. A significant association could be seen only in children below 20 kg with bilateral AOM when they were given insufficient dosages of amoxicillin. The OR for these children having a poor prognosis was 1.63 (95% CI 1.02–2.59, *P* = 0.04) in contrast to those who weighed more than 20 kg or with single-ear disease. Illness in autumn and winter had a strongly negative impact on AOM recovery. For physicians' behavior, underdosage was extremely common (89.1%), reflecting that the debates on dosage indeed exist even several years after the launch of the clinical practice guidelines. To a certain extent, our results were consistent with the latest guidelines revisions [21].

Many scholars questioned about guidelines recommendations being based on reports of bacterial drug resistance. Since laboratory bacteriological studies may be different from clinical trials with patients that evaluate a drug's curative effect, researchers suggest more studies evaluating clinical outcomes should be taken into consideration [14–17]. There was limited evidence based solely on “dosage.” This study is one of the few reports that simply investigated the relationships between amoxicillin dosage and clinical outcomes. Additional strength of our research was the precise determination of therapeutic effect. The treatment outcome (documented on medical records) regarding the eardrum status was evaluated by an otology specialist using video-telescopy coupled with a bright xenon light source which had been demonstrated to have the highest sensitivity (97.8%) and specificity (100.0%) [19].

Schrag's study in South America in 2001 concluded that treatment with short-course high-dosage amoxicillin (5 days, 90 mg/kg/day) reduced drug-resistant *Streptococcus pneumonia* (*S. pneumoniae*) with a probability of 8% and relative risk (RR) of 0.8 (0.60–0.97) compared with more days with a low dosage (10 days, 40 mg/kg/day). The short-course high-dosage amoxicillin was especially helpful for families with 3 or more children [10]. Garrison compared the curative effect of high-dosage (80–90 mg/kg/day) and standard-dosage (40–45 mg/kg/day) amoxicillin, finding that the two treatments had close rates of failure (11% versus 12%, *P* = 0.78). In addition, the side effects of medication, number of parents' inquiry calls, parents' subjective assessment of treatment days, and number of AOM reoccurrences were not significantly different for the two treatments [9]. Our results were relatively close to Garrison's research. However, the children enrolled herein were older than the above-mentioned two reports and only prescriptions with amoxicillin with clavulanate were included into analysis.

The clinical practice guidelines recommendation on amoxicillin dosage is controversial. The main reasons for the higher doses (80–90 mg/kg/day) in the guidelines are reports of penicillin-resistant *S. pneumoniae*. Before 2004, the prevalence rate of penicillin nonsusceptible *S. pneumoniae* (including intermediate and resistant strains) in the US had been increasing [22–24]. The resistance rate increased from 21% in 1995 to 25% in 1998 with a highest reported rate of 33%. Strain resistant to three or more antibiotics also increased from 10% to 14%. Therefore, the commonly used conventional amoxicillin dosage (40–50 mg/kg/day) was thought to be insufficient for controlling infection by drug-resistant bacteria. The committee therefore suggested that physicians should give higher doses of amoxicillin to AOM patients.

Previous studies had reported that 30% (15–50%) of dissociated *S. pneumoniae* from upper respiratory tract is not susceptible to penicillin, with a medium or high drug-resistance level. Only *S. pneumoniae *highly resistant to penicillin has no response to conventional dosages of amoxicillin (40–50 mg/kg/day). About half of nonsusceptible *S. pneumoniae* has a medium drug resistance level to penicillin (minimal inhibitory concentration (MIC): 0.1–1.0 *μ*g/mL), and the other half has a high drug-resistance level to penicillin (MIC ≥ 2.0 *μ*g/mL). Appropriately elevating dosages increase the drug concentration in middle ear fluid; theoretically, if this concentration surpasses the MIC of *S. pneumoniae* with a medium resistance level to penicillin, most cases of *S. pneumoniae* related AOM are responsive to amoxicillin treatment [25, 26].

As to other common pathogens in AOM, about 50% of *Haemophilus influenza (H. influenza)* and 100% of *Moraxella catarrhalis* (*M. catarrhalis*) are *β*-lactamase positive [25, 26]. In contrast to penicillin-resistant *S. pneumoniae*, AOM caused by* H. influenza* or *M. catarrhalis* requires a *β*-lactamase inhibitor such as clavulanic acid, a second- or third-generation cephalosporin, or other types of antibiotics. A sufficient dose of amoxicillin combined with clavulanic acid is effective for susceptible-to-medium-resistant *S. pneumoniae* and is also effective against *β*-lactamase-positive bacteria. For these reasons, the first choice of a majority of clinicians is oral amoxicillin with or without a *β*-lactamase inhibitor (unless patients are allergic to penicillin). In our hospital, a tertiary referral center as well as a teaching hospital, some of the specialists prescribed high-dose amoxicillin with clavulanate at the child's first visit unless patients were allergic to penicillin at the era of pneumococcal conjugate vaccination.

Another important factor that influenced prognosis was “season”. Illnesses in autumn and winter were constant strongly associated with a poor prognosis in this retrospective cohort study. Both univariate and multivariate analysis showed that outcome in autumn/winter patients was poorer than spring/summer patients (OR 2.47; 95% CI 1.17–5.23, *P* = 0.02). The result was even more obvious in children <20 kg (OR 4.80; 95% CI 1.82–12.67, *P* = 0.002). This result may be explained by a high prevalence for upper respiratory tract infections seen in autumn and winter, and people usually have several cold episodes. A child attends day care center or with siblings is likely to have multiple upper respiratory tract infections before resolution of a previous AOM, leading to more complicated disease course and poorer prognosis.

Reviewing the English literature, some reported that boys have poorer AOM prognosis than girls [27]. In our study, the ratio of boys who failed AOM primary control was lower than girls (21.3% versus 33.8%) but was not significant (*P* = 0.07). Female gender was not an independent risk factor for poor AOM control either in univariate or multivariate analysis (OR 1.61; 95% CI 0.77–3.36, *P* = 0.21). It had been reported that bilateral infection gave rise to poor prognosis [27]. Our study concluded that patients who had single-ear AOM initially had 19.8% failed control, as opposed to 33.3% for bilateral infection patients, although it was borderline significant (*P* = 0.05). This may be explained by insufficient samples (*n* = 165) in this research. Other parameters related to illness severity such as body temperature and earache, or infant crying, could not be comprehensively collected during the process of chart review and were thus not incorporated into outcome analysis.

Patients in medical centers might have different or complicated pathogens causing AOM more than patients from community groups, and the possibility of drug-resistant bacteria could be higher. Children enrolled in this study were from the ENT Department in our hospital, a medical center. Besides, the average age in our cohort was 4.88 years old, which is somewhat older than the commonly seen age for AOM. Older children should have a lower chance of AOM [28] because of relative maturity of the Eustachian tube-middle ear ventilation system; but on the contrary, patients who are over the common age and had middle ear infections might have more complex illnesses. These two perspectives could have led to the result of a comparable curative effect of regular-dose and high-dose amoxicillin with clavulanate.

In real-world settings, physicians prescribe antibiotics based on their understanding of the pathogenesis, course of disease, severity of the illness, pathogens causing the illness, and pharmacological knowledge. Other factors including physician's personal preference, bacterial drug-resistance reports from different areas or medical associations, caregivers' attitude, and insurance benefits may also play a role.

Our study has limitations. This is a retrospective cohort study; some of the possible risk factors such as past AOM history, previous visit to primary physicians, medication that had been used, day care attendance, siblings number, smoking in the household, and breast milk feeding could not be fully collected while doing chart review. The compliance for antibiotics was not known clearly either.

## 6. Conclusion

Our study demonstrated that high-dose amoxicillin with clavulanate as recommended in AOM clinical practice guidelines provided significant benefits only in children under 20 kg with bilateral illnesses. The question of how amoxicillin (amoxicillin-potassium clavulanate combination) dosage affects the prognosis of AOM needs more prospective controlled studies that comprehensively collect common prognostic factors with a large number of samples and laboratory bacteriological analyses.

## Figures and Tables

**Table 1:** Demographic data of 165 children.

Characteristic	Number (%)
Age: mean 4.91 ± 2.52 (y/o)	
Body weight: mean 19.36 ± 7.52 (kg)	
Gender	
Boy	94 (57.0)
Girl	71 (43.0)
Illness season	
Spring	52 (31.5)
Summer	28 (17.0)
Autumn	47 (28.5)
Winter	38 (23.0)
Single or bilateral disease	
Single-sided AOM	81 (49.1)
Bilateral AOM	84 (50/9)

Abbreviations: AOM: acute otitis media.

**Table 2:** Treatment outcome of acute otitis media.

	Overall		High dose		Underdose	
	*n*	(%)	*n*	(%)	*n*	(%)
Successful control	121	(73.3)	15	(83.3)	106	(72.1)
Eardrum normal	75	(45.5)	10	(55.6)	51	(34.7)
OME	70	(42.4)	5	(27.8)	55	(37.4)
Failed control	44	(26.7)	3	(16.7)*	41	(27.9)*

Sum	165		18		147	

Abbreviations: OME: otitis media with effusion. *Fisher exact test, *P* = 0.41.

**Table 3:** Factors relevant to poor outcome of acute otitis media (*n* = 165) by multivariate analysis (binary logistic regression model).

Variables	*P* value	OR of failed control (95% CI)
Girls	0.21	1.61 (0.77–3.36)
Weight*	0.48	0.98 (0.93–1.04)
Autumn/winter	0.02**	2.47 (1.17–5.23)
Bilateral AOM	0.09	1.96 (0.91–4.19)
Underdose	0.58	1.45 (0.38–5.53)

Abbreviations: AOM: acute otitis media; OR: odds ratio; CI: confidence interval. Reference groups in model: boys, illnesses in spring/summer, single-sided AOM, or high dose. *Age showed high collinearity with body weight in the model. Since antibiotics were given mainly based on body weight, weight was chosen instead of age in the regression model. **P < 0.05.

**Table 4:** Factors relevant to poor outcome of acute otitis media: subgroup analysis of children <20 kg (*n* = 110) and binary logistic regression model II.

Variables	*P* value	OR of failed control (95% CI)
Girls	0.84	1.10 (0.44–2.74)
Weight*	0.07	0.89 (0.78–1.01)
Autumn/winter	0.001**	4.90 (1.87–12.89)
Bilateral AOM and underdose	0.04**	1.63 (1.02–2.59)

Abbreviations: AOM: acute otitis media; OR: odds ratio; CI: confidence interval. Reference groups in model: boys, illnesses in spring/summer, single-sided AOM, or high dose. *Age showed high collinearity with body weight in the model. Since antibiotics were given mainly based on body weight, weight was chosen instead of age in the regression model. **P < 0.05.

## Abbreviations

AOM: Acute otitis media
CI: Confidence interval
*H. influenza*: *Haemophilus influenza*
*M. catarrhali*: *Moraxella catarrhalis*
MIC: Minimal inhibitory concentration
OME: Otitis media with effusion
OR: Odds ratio
*S. pneumonia*: *Streptococcus pneumonia.*
